# Exercise boost after surgery improves survival in model of metastatic breast cancer

**DOI:** 10.3389/fimmu.2025.1533798

**Published:** 2025-02-24

**Authors:** Rikke Stagaard, Adina Jensen, Tim Schauer, Marie Lund Bay, Ana Rita Tavanez, Sabrina Wielsøe, Merel Peletier, Jan Erik Strøbech, Victor Oginga Oria, Kamilla Westarp Zornhagen, Reidar Albrechtsen, Jesper Frank Christensen, Janine Terra Erler

**Affiliations:** ^1^ Biotech Research and Innovation Center (BRIC), University of Copenhagen (UCPH), Copenhagen, Denmark; ^2^ The Centre for Physical Activity Research (CFAS), Rigshospitalet, Copenhagen, Denmark

**Keywords:** physical exercise, metastasis, breast cancer, surgery, tumor microenvironment

## Abstract

**Introduction:**

Despite advances in breast cancer diagnosis and treatment of the primary tumor, metastatic breast cancer tumors remain largely incurable, and their growth is responsible for the majority of breast cancer-related deaths. There is therefore a critical need to identify ways to reduce metastatic tumor burden and increase breast cancer patient survival. While surgery and pharmacological treatments are the cornerstones of breast cancer intervention, epidemiological data suggests that physical activity can lower the risk of breast cancer development, improve adjuvant treatment tolerance, reduce the risk of disease recurrence and lower breast cancer-related death.

**Methods:**

In this preclinical study, we set out to examine the impact of exercise on metastatic development in triple negative breast cancer (TNBC), using different 4T1 metastasis models, voluntary wheel running and surgical interventions. Tumors were analyzed for hypoxia and immune cell infiltration.

**Results:**

Voluntary wheel running was observed to significantly increase metastasis-free survival, doubling the median survival time. However, these improvements were only observed when a boost in physical exercise occurred following surgery. To investigate this, we performed mock surgeries and confirmed surgical stress was needed to enable the positive effects of the boost in exercise on reducing metastatic tumor burden in mice with either spontaneous metastasis or experimentally-induced metastasis. These changes occurred in the absence of alterations in tumor growth, hypoxia and immune cell infiltration.

**Discussion:**

Taken together, our results suggest that having a boost of physical activity following surgery may be beneficial to delay breast cancer metastatic development.

## Introduction

Breast cancer has for the last three consecutive years been the most studied disease clinically (1), and it is also the most common cancer worldwide, despite mainly affecting women (99% vs.0.5-1% in men) (World health Organization (WHO), 2022). In fact, in 2022, 2.3 million women were diagnosed with breast cancer and 670.000 died due to the disease (2). Breast cancer is a heterogenous disease with at least four main subtypes, known as luminal A, luminal B, HER2-positive, and triple-negative breast cancer. The subtypes have different genetic, epigenetic, and clinical features and are classified according to their expression of certain receptors: estrogen receptor positive (ER+), progesterone receptor positive (PR+), human epidermal growth factor receptor positive (HER2+), and triple-negative (TNBC) that lacks all of the above-mentioned receptors (3).

The majority of breast cancer-related deaths can be ascribed to the development of metastatic disease (4, 5), which is notoriously difficult to treat due to the lack of effective treatment options (6). While there have been clear clinical advancements (7), metastatic breast cancer is generally considered incurable, and patients are thus only treated with the goal of prolonging survival and maintaining quality of life (8). Statistical analyses have revealed that approximately 1 out of 8 women will develop breast cancer (9) during their lifetime, and 20-30% of these patients will progress to develop incurable metastatic disease (10). Accordingly, there is a clinically urgent need to further the understanding of the development of metastasis and drive the development of better and more effective treatment options that can block or reduce metastasis.

While surgery and pharmacological treatments are the cornerstones of breast cancer intervention, physical activity also appears to have positive effects according to epidemiological data. In fact, the effect of exercise interventions has been examined in more than 292 breast cancer studies and in more than 20,808 patients (mainly with early-stage breast cancer) (11). Physical activity has been linked to lowering the risk of breast cancer development (12–14), improving quality of life after diagnosis (15, 16), mitigating fatigue and improving treatment tolerance during adjuvant treatment (16–18) and lowering the relative risk of overall death and breast cancer-related death (19–22). Despite a very large number of studies, only a few of these have examined the effect of exercise in patients with metastatic breast cancer (23), and of these the focus has mainly been on the impact of physical activity on quality of life and reduction of symptoms. However, promising clinical and observational studies have suggested that exercise can reduce the risk of disease recurrences for breast cancer (20) and increase survival in metastatic breast cancer (23).

In this preclinical study, we set out to examine the impact of exercise on metastatic development in triple negative breast cancer (TNBC) using a clinically relevant metastatic model with orthotopic tumor transplantation of breast cancer cells and subsequent surgical resection of the primary tumor to study the spontaneous development of advanced metastatic disease.

## Materials and methods

### Cell lines

The 4T1 murine breast cancer cell line was kindly gifted by Fred Miller (Wayne State University) and cultured at 37°C and 5% C0_2_ in Dulbecco’s modified Eagle medium glutaMAX (DMEM GlutaMAX; Gibco, Thermo Fisher Scientific, cat. no. 10566016, Grand Island, NY, USA) supplemented with 10% fetal bovine serum (Gibco, Thermo Fisher Scientific) and 1% penicillin-streptomycin (100 U/mL, Gibco, Thermo Fisher Scientific).

### Animals

8-15 weeks-old female BALB/cAnNRj mice were purchased from Janvier. All mouse experiments were conducted in compliance with the ARRIVE guidelines and approved by the Danish Animal Experiments Inspectorate (2015-15-0201-00656 and 2020-15-0201-00596). Mice were housed 2 mice/cage in standard housing cages with enrichment (nest materials, gnawing sticks, cardboard/plastic tunnels) and ad libitum food and water in a temperature and humidity-controlled room with a 12:12-h light-dark cycle. After arrival, the mice were acclimatized for at least a week. For exercise interventions, mice were giving access to running wheels (Starr Life Science, diameter 12 cm or Mouse Mag Wheels, The Columbus Instruments Starr Life Science, diameter 9.2 cm). The number of wheel rotations was monitored, and the running distances (km/mouse) calculated by converting wheel rotations to kilometers and dividing the results by two, since each cage contained two mice to prevent isolation-induced stress.

### *In vivo* cancer studies

Prior to all mouse studies, the cells were tested negative for mycoplasma.

**Figure 1:**
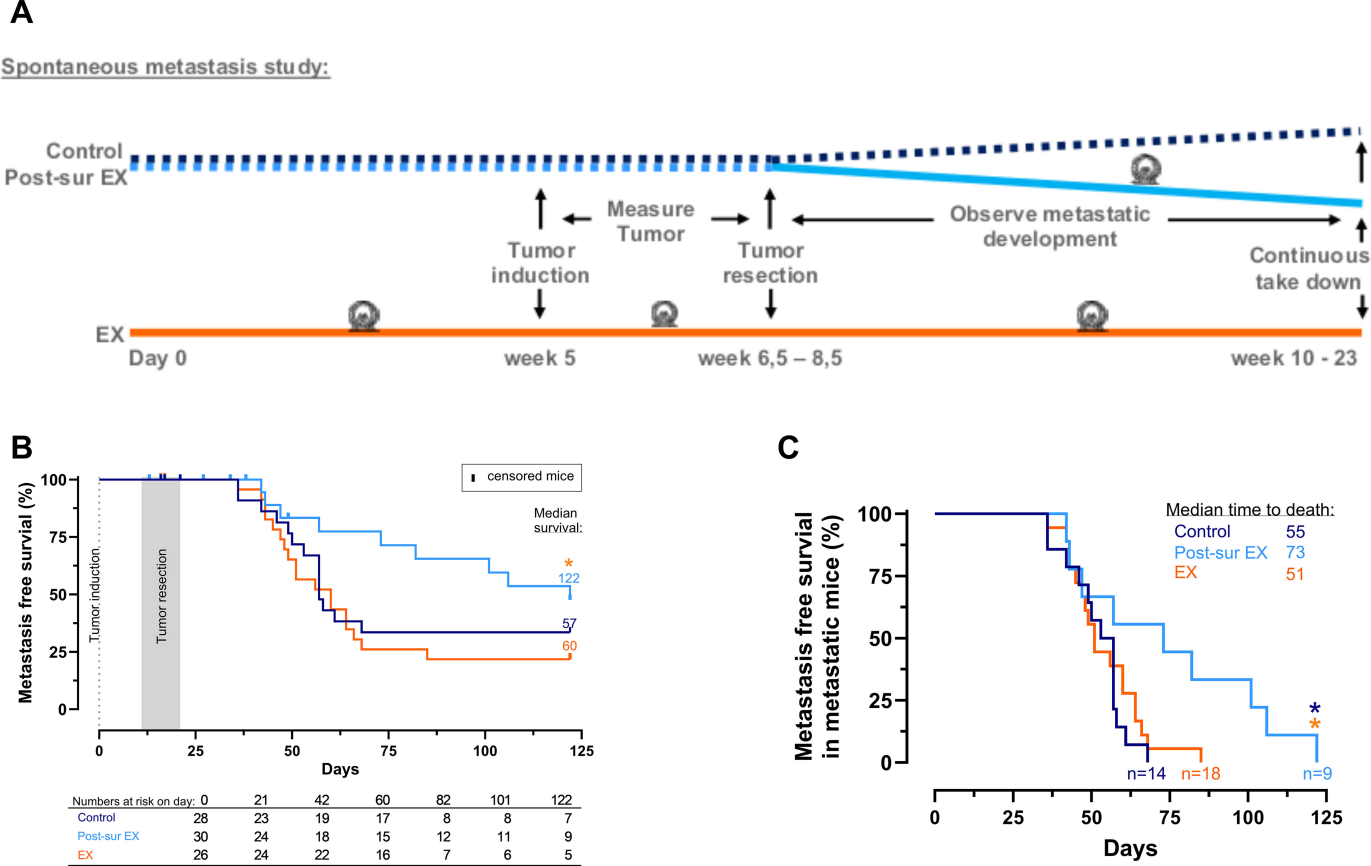
Exercise initiated after surgical tumor removal improves metastasis-free survival in an orthotopic model. **(A)** Visual representation of the experimental design. **(B)** The survival of mice subjected to surgical removal of mammary fat pad tumors with or without access to running wheels were analyzed with Kaplan-Meier analysis. The survival curves were compared using a Log-rank (Mantal-Cox) test. Pair-wise comparisons of the survival curves, revealed that the survival of the mice in the Post-sur EX group was significant longer than the EX group (p=0.03), while the rest of the pair-wise comparisons revealed no significant difference in survival between the groups (Control vs. Post-sur EX p= 019, Control vs. Ex p= 0.51). **(C)** The median time it took for mice to develop clinical signs of metastatic disease that necessitated euthanasia was examined by performing a Kaplan-Meier survival analysis combined with a Log-rank (Mantal-Cox) test on data from mice that developed metastatic disease. Pair-wise comparisons of the survival curves revealed that the development of metastatic disease in the mice in the Post-sur EX group was significant delayed compared to the Control group (p=0.02, dark blue *) and EX group (p=0.03, orange *).

For orthotopic tumor growth studies, mice were randomly assigned to 3 groups (n=14, repeated twice) and either got access to running wheels 5 weeks prior to a tumor induction (EX group), after primary tumor removal (Post-sur EX group), or not at all (Control group) (**Figure 1A**). Orthotopic tumors were induced by injecting 4x10^5^ cells in volume of 50 µl phosphate buffer solution (PBS)/mouse into the mammary fat pad and surgically resected once the primary tumors reached a size of 8-10 mm. 2-2.5% isoflurane was used to anesthetize the mice in both procedures, and prior to the tumor resection the mice were administered 0.05-1 mg/kg Buprenorphine subcutaneously and a mixture of lidocaine (5 mg/kg) and bupivacaine (1 mg/kg) around the tumor. Additionally, after the surgical intervention mice receive analgesia via the drinking water (6 mg/L Buprenorphine) for 48 hours. Tumor growth was determined by measuring the length and width of tumor and calculating volume using the following formula: volume (mm^3^) = (length (mm) * width (mm)^2^ * π)/6. An hour prior to resection, mice received an intraperitoneal injection with pimonidazole (60 mg/kg dosage using 10 mg/ml dilution, Hypoxyprobe™ Kit, Hypoxyprobe, Inc., HPI Catalog # HP1-XXX, Burlington, MA, USA).

The resected tumors were weighed, their volume determined and divided into pieces for further examinations. In a blinded manner, mice were assessed for clinical signs of metastasis and euthanized once the humane endpoints were reached. The lungs of mice were collected and weighed and processed for further examinations. Body weight was measured at set-up, tumor inoculation, tumor resection, 2-3 times weekly while monitoring for metastatic disease, and when euthanized. During the two studies, 20 mice had to be sacrificed due to surgical complications, wounds, or regrowth of primary tumors (n=6 (Control), n=10 (Post-sur EX group), and n=3 (EX group)). These mice were as a result censored from the survival analysis.

**Figure 2:**
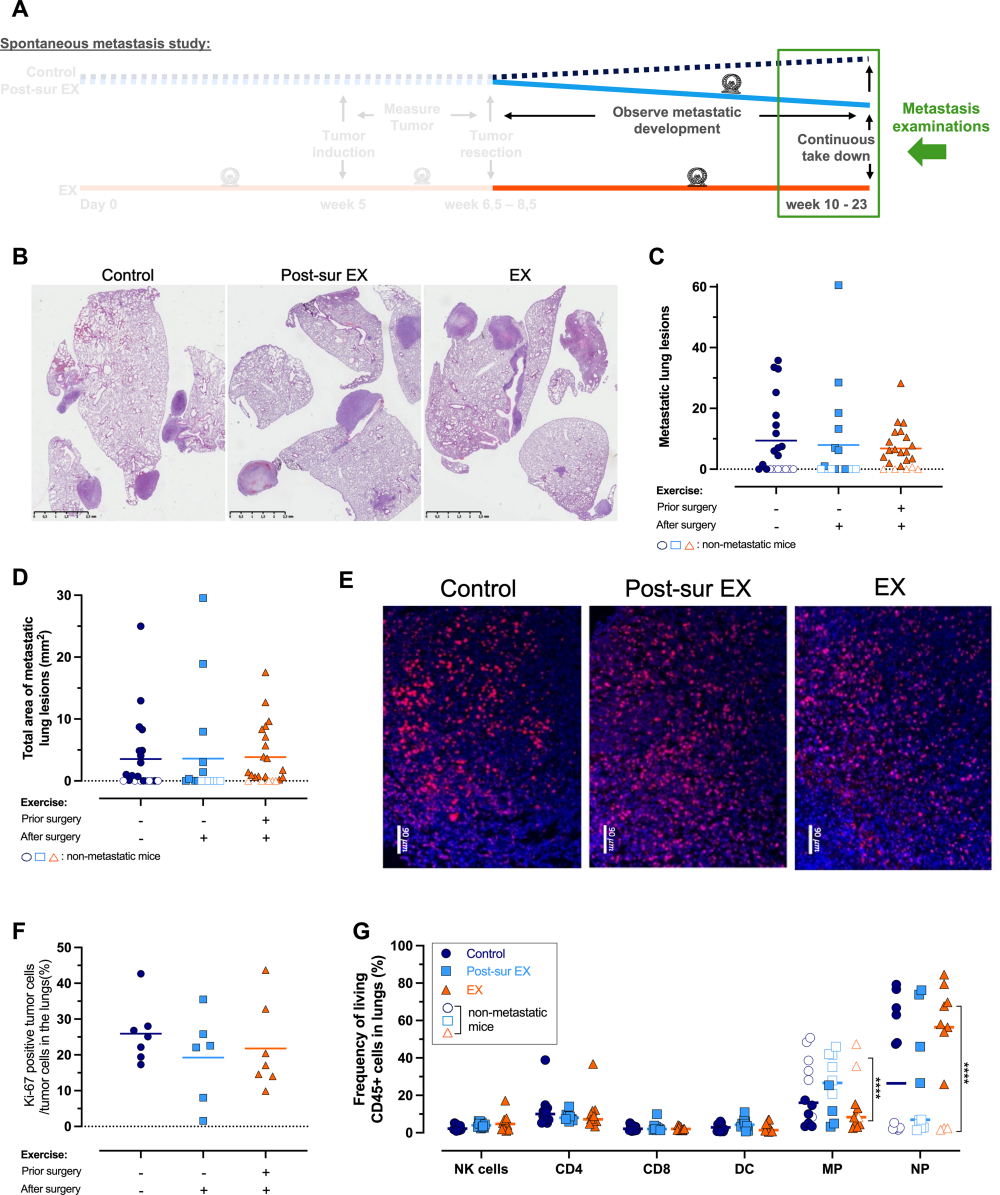
Analysis of lung metastases reveals no significant differences between the groups in orthotopic model. **(A)** Visual representation of the experimental design and highlighting the focus of the data – i.e. examinations of collected lungs. **(B)** Representative images of the degree of lung metastasis. **(C)** Number of metastatic lung lesions (Control= 21, Post-sur EX= 17, EX= 22). **(D)** Total area of metastatic lung lesions. **(E)** Representative images of Ki-67 positive stained tumor cells in the lungs of mice **(F)** Percentage of Ki-67 positive stained tumor cells per cells in the lungs (Control= 7, Post-sur EX= 6, EX= 7). **(G)** Flow cytometry analysis of the immune landscape in the collected lungs (Control= 12, Post-sur EX= 10, EX= 11). NK = natural killer cells, CD4 = CD4 positive T cells, CD8 = CD8 positive T cells, DC= dendritic cells, MP= Macrophages, NP=Neutrophils. Parametric data was analyzed by a one-way ANOVA paired with Holm-Šídák’s multiple comparisons test (**F**, **G**: DC) and nonparametric data with a Kruskal-Wallis test with Dunn’s multiple comparisons test (**C**, **D**, **G**: NK cells, CD4, CD8, MP, and NP). A two-way ANOVA was used to analyzed the difference in MP and NP between mice with and without metastatic disease **(G)**.

For experimental lung metastasis studies mice were randomly assigned to 4 groups (n=8, repeated twice) and either got access to running wheels 5 weeks prior to metastasis induction (EX groups, Pre EX group), after metastasis induction (Post EX group), or not at all (Control group) (**Figure 2A**). Lung metastasis was induced by injecting 3x10^5^ cells in volume of 200 µl phosphate buffer solution (PBS)/mouse into the tail vein. Mice were weighed and euthanized after 12 days, where their lungs were collected and weighed.

For surgical stress studies, mice were randomly assigned to 3 groups (n=6, repeated three times). All groups were induced with metastatic disease (3x10^5^ cells in volume of 200 µl PBS/mouse injected into the tail vein), but while the Control and Post-sur EX groups underwent surgical resection of the tissue around the 4^th^ mammary fat pad to mimic a tumor resection, the sham group was only anaesthetized. The mice received the same anesthesia and analgesia as previously described. A day after the surgery the EX group got access to running wheels (**Figure 2A**). Mice were weighed and euthanized 14 days after 4T1 inoculation, where their lungs were also collected and weighed. All mice used for the manuscript were euthanized by cervical dislocation or decapitation.

### Histology and immunohistochemistry

Part of the tumor tissue and isolated lungs tissue were fixed in 10% neutral buffered formalin overnight at 4 °C and then processed for standard paraffin embedment. Paraffin-embedded tissue was sliced to 5 µM tissue slides and mounted on glass. All lung slides were stained with hematoxylin and eosin, scanned on a Hamamatsu NanoZoom slide scanner, and quantified for metastatic disease using NDP.view2 software by determining the number and size of the metastatic lung lesions.

Additionally, immunohistochemistry stainings were conducted on tumor (n=12-13/group) and lung sections (n=6-7/group) from the orthotopic tumor studies, where the tissue sections were de-paraffinized, rehydrated, and heated with antigen retrieval buffer.

After the antigen retrieval treatment, the tumor section was stained to assess tumor hypoxia via the hypoxia marker pimonidazole hydrochloride, which mice were injected with one hour prior to having their tumor removed. The slides were washed in water, incubated for 10 min with 3% H_2_O_2_, and washed in Tris-buffered saline with tween (TBS-T). The tumor sections were encircled with a waterproof pen, blocked for 60 min, and incubated overnight with the primary antibody, i.e. anti-pimonidazole mouse IgG1 monoclonal antibody (H Hypoxyprobe™ Kit, Hypoxyprobe Inc.) at 4°C. The tissue sections were then washed in TBS-T buffer, and incubated for 45 min at RT with the DAKO HRP Mouse antibody (Agilent Technologies), followed by a washing step with TBS-T buffer and the addition of DAB Chromogen (Peroxidase) (Vector Laboratories) as per kit instructions. Lastly, the tissue sections were counterstained with Mayer’s hematoxylin (Sigma-Aldrich), dehydrated, mounted with DPX, and scanned on the Hamamatsu NanoZoomer-XR (Hamamatsu) whole slide scanner (×40 magnification). Tumor hypoxia was quantified via ImageJ (24) by manually measuring the total area of both tumor and hypoxia and calculating the fraction of the tumor affected by hypoxia.

The lung sections were stained for Ki-67, as Ki-67 is a prognostic marker associated with breast cancer cells’ proliferative potential and an indicator of prognosis (25). Following antigen retrieval, the lung sections were washed with PBS, encircled using a PAP pen (Dako, Denmark), incubated with 5% donkey control serum (D9663, Sigma-Aldrich, Merck Life Science A/S, Denmark) for 10 min at RT, incubated with primary antibody against Ki-67 (1:1,000-1:2000, ab15580; Abcam, San Diego, USA) at 4 °C overnight. The next day, the sections were washed twice in PBS and incubated with secondary antibody using fluorescence Donkey anti rabbit Alexa 546 for 1 hr at RT (Invitrogen, Taastrup, Denmark, diluted in PBS 1:1000) and counterstained with DAPI before being mounted. Images of the slides was captured with a x40 magnification on a Zeiss Axiovert 220 Apotome system. The images were processed using the Axiovision program (Carl Zeiss) and all images were imported, and the threshold was set for all. The MetaMorph microscopy automation and the ImageJ analysis software (24) were used for automatic nuclei counting and for detection of Ki-67 stained tumor cells. The total number of DAPI stained tumor cells was estimated by automatic nuclei counting. The number of Ki-67 stained cells were counted manually and the fraction of tumor cells expressing the Ki-67 antigen was determined.

The quantifications (metastatic lesions, hypoxia, Ki-67 stainings) were performed while blinded.

### Flow cytometry

Frozen and digested tumor and lung tissue were washed once in FACS buffer (PBS + 2% FBS) and incubated with FC-block and live-dead staining for 15 minutes at RT. Afterwards, cells were washed once, followed by antibody staining (**Supplementary Table 1**) for 30 minutes at 4°C and followed by fixation in 2% paraformaldehyde for 10 minutes at RT. Cells were stored in FACS buffer until acquisition within the next 3 hours.

Receptor surface expression was acquired using an LSRFortessa equipped with 3 lasers (488nm, 640nm, 405nm) maintained by the flow cytometry core facility at Copenhagen University using FACSDiva software v.8.01 (BD Biosciences, USA). Analyses of blinded samples (gating strategy in **Supplementary Figure 1**) and compensation were performed in FlowJo v.10.6.1 (BD Biosciences, USA). Gating was based on Fluorescence minus one (FMO) controls for each parameter. Cells were defined as follows: all cells (DAPI^-^, CD45^+^), T cells (CD3^+^), CD4 T cells (CD3^+^ CD4^+^), CD8 T cells (CD3^+^ CD8^+^), NK cells (CD3^-^ CD24^-^ SSC^low^ CD49^dim/hi^ Nkp46^dim/hi^), Neutrophils (Ly6G^+^), Macrophages (Ly6G^-^ SSC^hi^ F4/80^+^) and Dendritic cells (Ly6G^-^ F4/80^-^ MHC-II^+^ CD11c^+^ CD11b^+/-^).

### Analysis and statistics

Statistical analyses were performed in Prism 10 (version 10.2.2). Statistical significance was defined as a p-value < 0.05 throughout. The survival of mice subjected to surgical removal of mammary fat pad tumors with or without access to running wheels was analyzed with Kaplan-Meier analysis. The survival curves were compared using a Log-rank (Mantal-Cox) test. The median time it took for mice to develop clinical signs of metastatic disease that necessitated euthanasia was examined by performing a Kaplan-Meier survival analysis combined with a Log-rank (Mantal-Cox) test on data from mice that developed metastatic disease with “Pair-wise” comparisons of the survival curves. To examine if the tumor growth of mice without access to running wheels differed from mice with access, the data was log transformed with a natural logarithm and analyzed by fitting a nonlinear regression model using the exponential growth with log(population) equation. The analysis revealed that one curve fitted both data sets (i.e. sedentary and exercising mice). For analyses of two groups, parametric data was analyzed with an unpaired t-test or Welch's t test, while nonparametric data was analyzed with a Mann Whitney test. When analyzing the difference between multiple groups, parametric data was analyzed with an ordinary one-way ANOVA paired with Holm-Šídák’s multiple comparisons test/Šídák’s multiple comparisons test and nonparametric data with a Kruskal-Wallis test with Dunn’s multiple comparisons test. Furthermore, a two-way ANOVA was used to analyze the difference in macrophages and neutrophils between mice with and without metastatic disease.

## Results

### Physical exercise did not affect food intake or body weight in an orthotopic model of TNBC

We investigated the effect of voluntary exercise on tumor development and metastasis in a group of immunocompetent mice with induced TNBC. We used 4T1 murine breast cancer cells due to the high clinical relevance of the experimental model, which includes easy orthotopic transplantation in the tissue of origin (mammary fat pad) and spontaneous development of metastatic disease with tumor cell dissemination patterns similar to that of human mammary cancer (26). Specifically, we combined an orthotopic tumor transplantation with a subsequent surgical removal of the primary tumor to mimic the clinical setting, where primary tumors are surgically removed, while potential metastatic lesions remain, and to allow the development of metastatic disease to become the experimental endpoint (27).

Mice were given access to running wheels either 5 weeks prior to tumor induction with syngeneic 4T1 breast cancer cells (EX group), or after primary tumor removal (Post-sur EX group), or not at all (Control group) (**Figure 1A**). We observed no differences in the running distance between the different exercise groups (**Supplementary Figures 2A, B**), nor differences in the food intake or body weights across all groups as recorded throughout the experiment (**Supplementary Figures 2C, D**, respectively).

### Exercise-increased survival occurs in the absence of changes in hypoxia and the immune landscape at the primary tumor

Exercise did not significantly affect the primary tumor growth rate (**Supplementary Figure 3B**). Furthermore, there was no significant difference in the time it took for the tumors to reach a size that required their removal (**Supplementary Figure 3C**), nor in the weight of the resected primary tumors (**Supplementary Figure 3D**).

In contrast, we observed a striking difference in survival, whereby the introduction of voluntary wheel running after surgical removal of the primary tumor led to increased median survival of the mice (**Figure 1B**), which was significantly different from the mice with continuous access to running wheels (p= 0.03). Furthermore, the median time it took for mice to develop clinical signs of metastatic disease that necessitated euthanasia was significantly delayed in the Post-sur EX group compared to both the Control group (p=0.02) and the EX group (p= 0.03) (**Figure 1C**).

It is known that hypoxia at the primary tumor can influence metastasis and survival (28) and could likely be reduced by exercise (29). We, therefore, analyzed the resected tumors for hypoxia, but observed no significant differences (**Supplementary Figures 3E, F**). Wennerberg et al. and Garritson et al. previously indicated that voluntary wheel running could lead to a more favorable immune landscape in mice with 4T1 breast cancer by reducing (30) or delaying (31) immune suppression and increasing the activation of NK cells and CD8^+^ T cells (30). We therefore examined, if the differences in metastasis-free survival could be explained by alterations in the immune landscape of the primary tumors by flow cytometry (**Supplementary Figure 1A**). However, we detected no significant differences in immune cell infiltration between the EX group and the Control/Post-sur EX group (**Supplementary Figure 3G**).

### Exercise-increased survival occurs in the absence of changes in metastatic tumor burden and immune landscape at the time of termination

Next, we examined the lungs of the mice at the time of termination, as it is the first site of metastatic spread in both human patients (22-77%) and mice transplanted with 4T1 cells (95%) (26). We detected no differences between the groups with regards to the number (**Figures 2B, C**) or the total area of metastatic lung lesions (**Figure 2D**). Therefore, once the mice developed metastatic disease, the endpoint metastatic burden appeared to be similar between the groups. Consistently, there was no change in Ki-67 expression (**Figures 2E, F**), a proliferation marker that is associated with worse disease-free survival and overall survival in patients with resected TNBC (32, 33).

We performed flow cytometry analysis of the lungs (**Supplementary Figure 1B**), and observed no significant differences in NK cells, CD4^+^ and CD8^+^ T cells, dendritic cells, macrophages, or neutrophils between the groups (**Figure 2G**). However, when we compared the mice with metastatic disease to mice without metastatic disease, we noted a significant decrease in the frequency of lung macrophages and increase in the frequency of lung neutrophils in the mice with metastatic disease (**Figure 2G**).

### Exercise alone does not affect metastatic tumor burden in an experimental metastasis model

**Figure 3:**
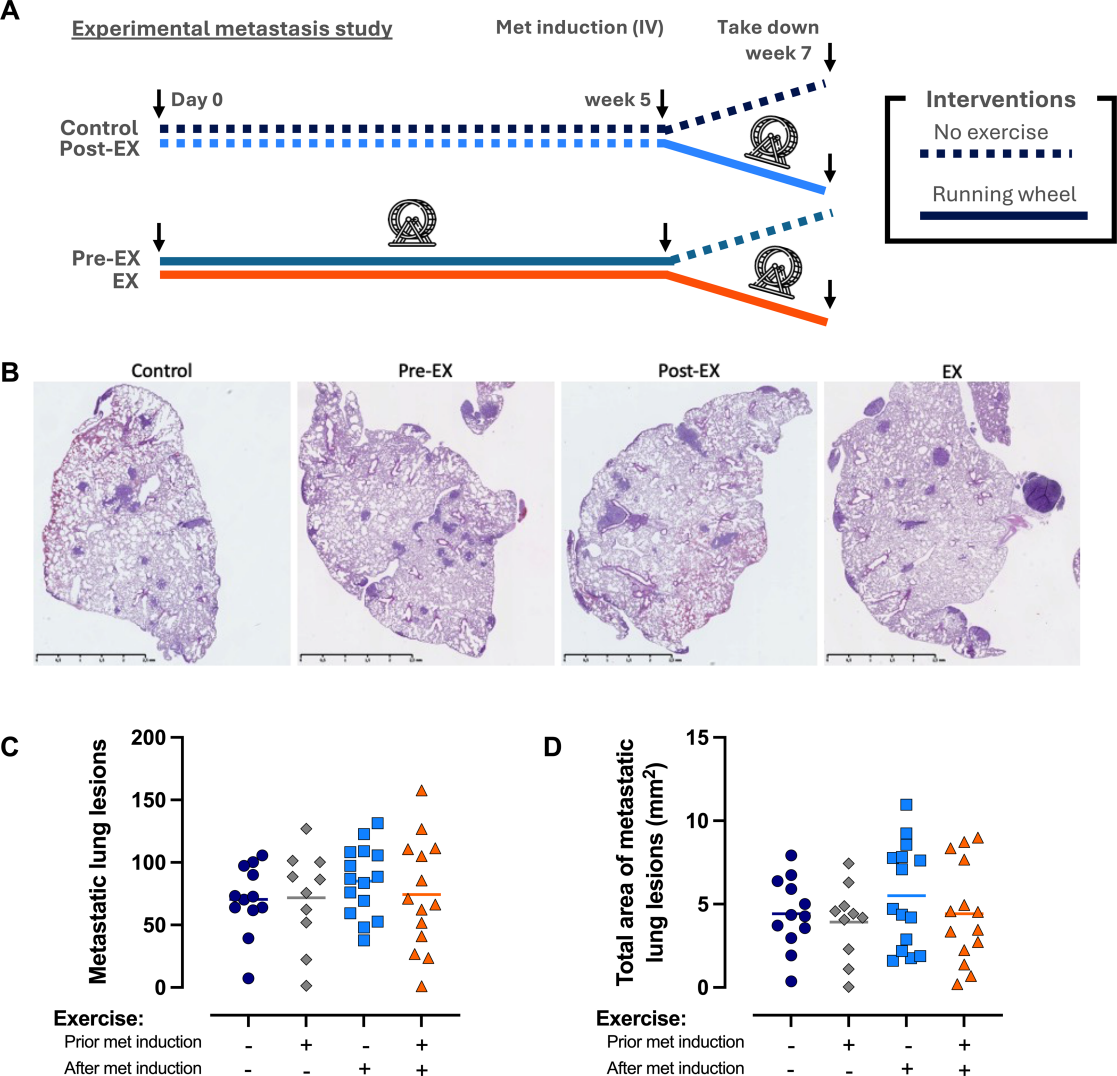
A boost of exercise alone does not affect the development of metastatic disease in an experimental metastasis model. **(A)** Visual representation of the experimental design. **(B)** Representative images of the degree of lung metastasis. **(C)** Number of metastatic lung lesions. **(D)** Total area of metastatic lung lesions. The data in C and D were analyzed by a one-way ANOVA paired with Šídák’s multiple comparisons test (Sedentary= 12, Pre-EX=10, Post-sur EX= 15, EX= 14).

We speculated that the lack of differences between the groups could be explained by the fact that we only compared them at a timepoint, where all the mice were deemed to have clinical signs of metastatic disease that necessitated euthanasia. We, therefore, performed a new experimental metastasis study with intravenous (IV) injections of 4T1 cells, where mice either had access to a running wheel throughout the experiment, prior to induction of metastasis, after the induction of metastasis, or not at all (**Figure 3A**). Furthermore, instead of having a continuous take down, the study was concluded on day 14 and the degree of metastatic disease in the lungs of all mice was evaluated by histological examinations of lungs sections. We observed that mice who ran prior to metastasis induction had a higher running distance (**Supplementary Figures 4A, B**), that food intake was significantly increased in mice that had access to running wheels compared to mice without (**Supplementary Figures 4C, D**), and that body weights of the mice increased throughout the experiment (**Supplementary Figure 4E**). However, to our surprise, we again saw no differences in the number of metastatic lung lesions between the groups (**Figures 3B, C**) or the total area of metastatic lung lesions (**Figure 3D**). In this setup, exercise alone did not abrogate metastatic development.

### The antimetastatic effect of a boost in exercise seems dependent on the presence of surgical stress

**Figure 4:**
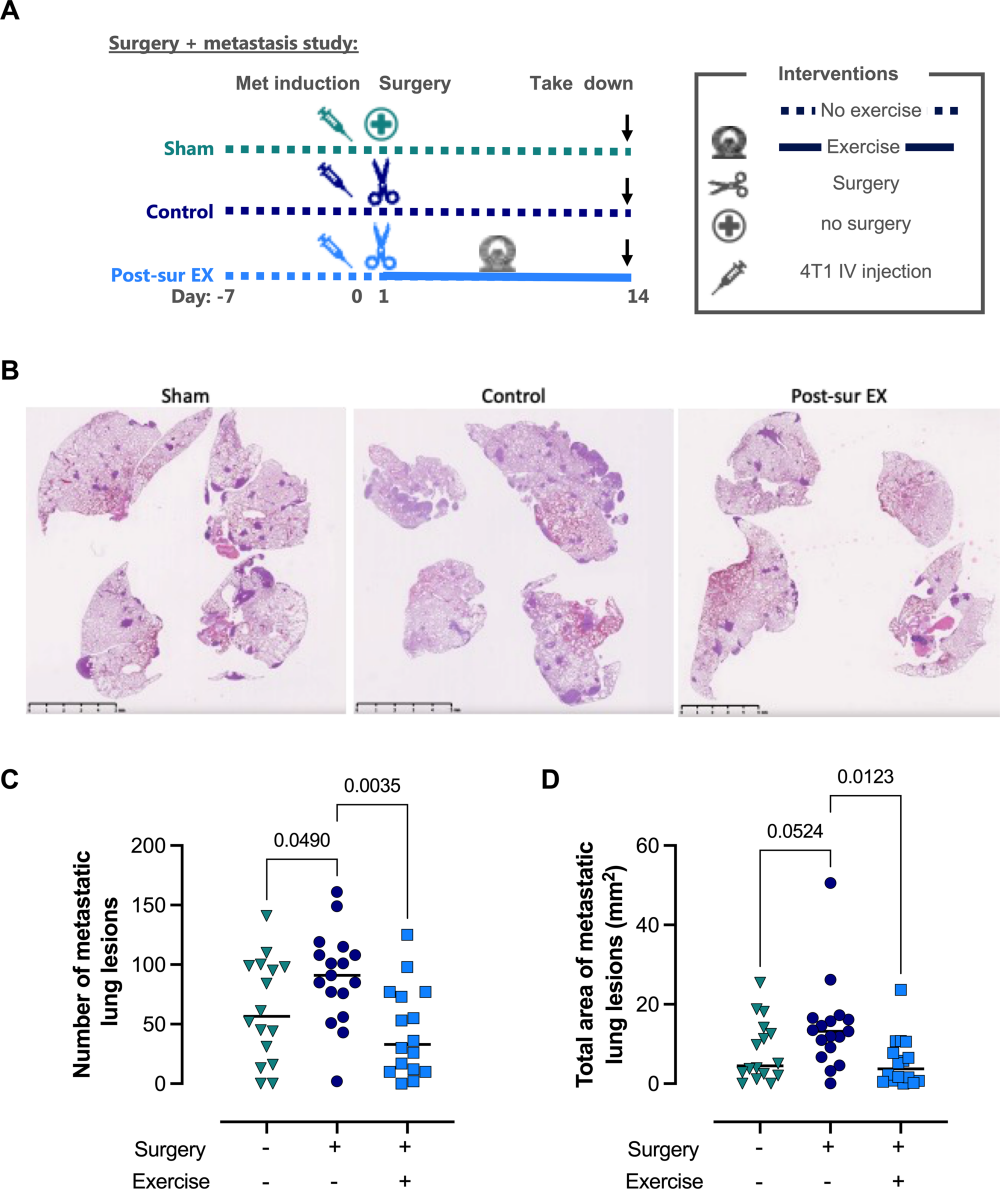
Initiating exercise following surgery significantly reduces development of metastatic disease. **(A)** Visual representation of the experimental design. **(B)** Representative images of the degree of lung metastasis. **(C)** Number of metastatic lung lesions. **(D)** Total area of metastatic lung lesions. The data were analyzed with an ordinary one-way ANOVA paired with Holm-Šídák’s multiple comparisons test (Sham= 16, Control= 17, Post-sur EX= 16).

Given that the orthotopic model also included a surgery, we postulated that this could promote the exercise-mediated increase in metastasis-free survival. Of note, surgical stress has been shown to exacerbate metastatic disease in both animal models and cancer patients (34, 35). We, therefore, performed another IV experimental metastasis study, which included a mock-surgery to induce surgical stress. Specifically, all mice were induced with metastatic disease, and then either only anaesthetized or also subjected to a skin removal surgery similar to that of a primary breast tumor resection. Half of the mice that underwent surgery were given access to running wheels the day after surgery, while the rest were not. After 14 days all mice were taken down and their lungs examined for metastatic disease (**Figure 4A**). When we compared the number of lung lesions in the group undergoing surgery to the group that was only anaesthetized, we observed that the surgery alone group had significantly more metastatic lung lesions (**Figures 4B, C**). However, the number of metastatic lung lesions was significantly reduced if the mice had access to a running wheel following undergoing surgery (**Figures 4B, C**). A similar pattern was observed when we assessed the total area of metastatic lung lesions, such that surgery alone increased the affected lung area. However, access to the running wheel after surgery significantly reduced the total lung area affected by metastatic lesions (**Figure 4D**). These results suggest that the increase in metastasis-free survival observed in the orthotopic model was due to a boost of exercise occurring after but dependent on surgery.

## Discussion

To our knowledge, our study is the first to preclinically examine the effect of exercise on metastatic development in a clinically relevant setting, where the primary tumor was surgically removed, while potential metastatic lesions remained (26). This allowed us to examine how continuous voluntary wheel running affected the development of metastatic disease, as well as the effect of only initiating exercise after primary tumor resection. Our primary finding was that initiating voluntary wheel running after removal of the primary tumor significantly increased the metastasis-free survival and doubled the median survival time. However, interestingly, we found no beneficial effect of exercise (regardless of when it was initiated) once mice exhibited clinical signs of spontaneous metastatic disease. Specifically, once mice were euthanized due to metastatic disease, we observed no differences between the groups regarding the number of lung lesions, the total lung area affected by metastasis or the presence of Ki-67 positive tumor cells in the lungs. Furthermore, exercise alone was not detected to have a beneficial effect on metastatic development in an experimental model for metastasis, where metastasis was induced with IV injections. However, exercise did reduce the number of metastatic lung lesions in this model, if the mice also underwent surgical stress the day after inducing metastasis. Taken together, our results indicate that initiating a boost of physical activity (as mice are naturally quite active) is beneficial following surgery and can delay metastatic development in mice with either spontaneous or experimentally-induced metastasis.

Epidemiological studies have highly suggested that exercise has a beneficial effect on breast cancer. However, most studies have not examined whether the effect was dependent on the molecular subtype of cancer. To understand the effect of exercise in a breast cancer setting this should be considered, as breast cancer is not one uniform disease, but rather a heterogeneous group of diseases that differ from one another regarding histology, genomic alterations, gene expression, hormone status, metastatic behavior, and treatment responses (36–38). In our study, we examined how exercise affected both TNBC development and metastasis. From a clinical perspective, the effect of exercise in this population is still not clear. For instance, while Delrieu et al. found a beneficial impact of physical activity on the overall survival of patients with metastatic breast cancer, a subgroup analysis revealed that physical activity was only associated with a statistically significant improved overall survival in the HER2 positive subgroup, but not in luminal metastatic breast cancer or TNBC (23). Furthermore, a meta-analysis by Ibrahim et al. reported that while post-diagnosis physical activity was shown to reduce breast cancer deaths by 34% and disease recurrence by 24%, this beneficial effect only seemed to involve women with estrogen receptor (ER) -positive breast cancer (39). In contrast, the Shanghai Breast Cancer Survival Study, a prospective cohort study, showed that regular postdiagnosis exercise was associated with a lower risk of all-cause mortality and recurrence/disease-specific mortality in women with ER and progesterone receptor-negative breast cancer (40). In addition, data from the NIBBLE study, the Women’s Health Initiative, and the California Teachers Study indicated that physical activity was associated with a reduced risk of developing TNBC (41–43). Similarly, no clear consensus has been found regarding the effect of exercise on metastatic disease in preclinical research, potentially due to a wide methodological heterogeneity. In fact, the published preclinical experimental data is conflicting (23, 24), something we also observed, as we, despite using large group numbers, still saw variation and even observed an opposite effect in the pilot experiment for the mock surgery setup. This highlights the need for further research to elucidate the underlying molecular mechanisms for the potential positive effect of physical activity on metastatic development and underlines the importance of performing multiple repeats of experiments to elucidate the real effect/trend. Especially, as experimental results are also contradictory even when the same cancer model (4T1) and exercise modality (voluntary running wheels) is used, as subjecting 4T1 tumor-bearing mice to wheel running has been found to both promote metastasis (44), not affect metastasis (45), and non-significantly reduce metastasis (30, 31). However, no studies as of yet included surgery as part of the setup.

Previous preclinical studies have suggested that exercise exerts its beneficial anticancer effect by recruiting and activating different immune cells (46). For instance, an exercise-mediated 4T1 tumor growth suppression vanished when examined in T-cell deficient mice (47), while NK-cells (but not T-cells) proved essential for the exercise-mediated tumor growth control in mice challenged with B16F10 tumors (48). However, flow cytometry analysis in our study presented here showed no difference in the immune landscape in the lungs of mice with metastatic disease regardless of whether they had access to running wheels or not. The lack of difference could potentially be explained by the timing of the analysis. The lungs of the mice were only examined once the mice had clinical signs of metastatic disease and thus had reached the humane endpoint. Perhaps a difference could have been seen, if lungs were collected at an earlier timepoint of 1-2 weeks after the surgery and then assessed for the composition of the immune cells.

Our results did not elucidate a clear mechanism of action that could explain why initiating exercise after surgery limited metastatic development or why the same effect was not seen in mice that had continuous access to running wheels. The fact that the average running distance of mice in the Post-sur EX group and the EX group was similar, does however indicate that the groups had similar exercise compliance, and thus that this did not contribute significantly to the observed survival outcomes. However, because of the lack of effect in mice that continuously exercise, we hypothesize that the beneficial effect could be mediated by the body’s adaptation to exercise during the critical perioperative period. We expect this could be the case, because not only does exercise have a multitude of effects on the body by initiating interaction and crosstalk between multiple organs, tissues, and regulatory systems, including the immune system and the metabolism (49); the complex physiological response to exercise also differs between untrained individuals adapting to exercise and trained individuals (50, 51). It is therefore likely that the impact of exercise adaptation in the critical postoperative period of a tumor resection would differ between trained and untrained mice, and thus that initiating voluntary wheel running in the two groups after surgery could lead to different impacts on tissues, regulatory systems, the immune system, and the metabolism. The immune landscapes and systemic immune response could differ at an earlier timepoint. Furthermore, if the adaptation to exercise in the untrained mice post-surgery resulted in an altered metabolism, that could also play a part, as it is well known that the metastatic process and metabolic pathways a highly intertwined (52). For instance, exercise has been suggested to reprogram the metabolic needs of distant organs and thereby increase their resistance to metastatic development (53).

Surgical resection of solid tumors is a necessary procedure for most cancer patients and has undeniable prognostic benefits (54). Still, the perioperative period is deemed critical, as a growing amount of evidence suggest that surgeries elicit a surgical stress response and/or surgical complications that promote postoperative metastatic spread and/or disease recurrence by activating and increasing the growth of pre-existing dormant micrometastases or residual cancer cells at the surgical site (34, 55–60). This surgical stress response has thus been linked to the development of metastatic disease in both animal models and cancer patients and is believed to be caused by postoperative dysfunction of NK cells, potential dissemination of cancer cells from the primary tumor, induced local and/or systemic inflammation, and immune suppression (34, 35, 58, 61–69). The postoperative period is therefore an exceptionally vulnerable time for the development or growth of metastases (35). Therefore, it not only represents an ideal moment to therapeutically target the metastatic process, but also a window of opportunity, where exercise could have an anti-metastatic effect, especially when considering that exercise mobilizes key effector cells of the immune system and reduces inflammation, including increasing the number of circulating NK cells, their cytotoxicity, and activation (30, 46–48) (70). We therefore believe that it is very plausible that the beneficial effect observed in the Post-sur EX group, is mediated by an increased recruitment and activation of NK cells compared to the EX group. Particularly when considering that NK cells control micrometastatic disease (48, 71), their cytotoxicity is an independent prognostic marker for overall survival in patients with metastatic disease (72), and because an association between low NK levels during the post-operative period and a higher rate of cancer recurrence and mortality has been observed (67, 68). Furthermore, the effect in the Post-sur EX group could also perhaps be mediated by a dampening of the local inflammatory wound response and systemic inflammation caused by surgery, as exercise is known to have anti-inflammatory effects (73, 74). This could be important, as several *in vivo* studies have indicated that surgery induced inflammation and subsequent increase in growth factors and proangiogenic compounds can increase the risk of cancer recurrence by reactivating dormant micrometastases (34). In fact, the degree of surgery-induced inflammation seems to correlate with the number of lung metastasis in a metastatic mouse model (75). Future studies will examine the role of exercise in connection with surgical stress and how exercise affects the postoperative NK cell dysfunction, inflammation, the immune system, and metastasis.

In conclusion, we examined the effect of exercise on metastatic development in different metastatic models for TNBC. Voluntary wheel running was observed to reduce the number of metastatic lung lesions or significantly increase the metastasis-free survival and doubled the median survival time, but only in settings where the mice underwent a surgery and initiated a boost of exercise after the surgery. Taken together, our results therefore indicate that initiating exercise and thus having a boost of physical activity is beneficial following surgery and can delay metastatic development in mice with either spontaneous or experimentally-induced metastasis. Even though we only observed a beneficial anti-cancer effect of exercise if it was initiated after a surgery, we are not advocating for only initiating exercise there. Our wish is to focus attention to the post-operative period and highlight it as a great window of opportunity to counteract metastasis. Exercise could be one way to do so, especially as exercise already has gained a prominent role in clinical oncology due to its’ abundant supporting care and health benefits, including improving quality of life, maintaining muscle strength during therapy, reducing treatment-related complications and improving survival outcomes of cancer patients (46, 76–79).

## Data Availability

The original contributions presented in the study are included in the article/**Supplementary Material**. Further inquiries can be directed to the corresponding author.

## Supplementary material

Supplementary Figure 1 Gating strategies for flow cytometry. **(A)** Gating strategy for panel 1. Cells were defined as T cells (CD3+), CD4 T cells (CD3+ CD4+), CD8 T cells (CD3+ CD8+), NK cells (CD3- CD24- SSClow CD49dim/hi Nkp46dim/hi). **(B)** Gating strategy for panel 2. Cells were defined as Neutrophils (Ly6G+), Macrophages (Ly6G- SSChi F4/80+) and Dendritic cells (Ly6G- F4/80- MHC-II+ CD11c+ CD11b+/-).

Supplementary Figure 2 Health data from the orthotopic tumor growth studies reveals no significant differences between the groups. **(A)** The collected running distance of the exercising mice, shown as median of the groups (solid lines) with indication of the SD (transparent areas) (orange = EX, light blue = Post-sur EX). **(B)** Average running distance of the exercising mice **(C)** Average food intake. **(D)** Body weight of the mice a study start, tumor inoculation (Tumor), tumor resection (Resection), and at study end/humane endpoints (Study end).

Supplementary Figure 3 Analysis of primary tumors reveals no significant differences between the groups in orthotopic model. **(A)** Visual representation of the experimental design and highlighting the focus of the data – i.e. examinations of resected tumors. **(B)** The tumor growth of mice with or without access to running wheels (Control/Post-sur EX= 58, EX=26). The solid line represents mean tumor growth, while the shaded area between the stippled lines constitutes the SD. Tumor growth data was analyzed with a nonlinear regression model using the exponential growth with log(population) equation, but not significant difference was detected. **(C)** Time from tumor inoculation to resection. **(D)** Weight of resected tumors. **(E)** Representative images of the degree of tumor hypoxia determined. **(F)** Percentage of tumor tissue affected by hypoxia (Control/Post-sur EX= 24, EX=13). **(G)** Flow cytometry analysis of the immune landscape in the resected tumors (Control/Post-sur EX= 28, EX=13). Parametric data was analyzed with an unpaired t-test (Figure C, G: T cells, MP) or a Welch's t test (Figure G: DC) and nonparametric data with a Mann Whitney test (Figure C, F, G: NK cells, CD8, NP). Tumor growth data was analyzed with a nonlinear regression model using the exponential growth with log(population) equation. (NK cells = natural killer cells, CD8 = CD8 positive T cells, DC= dendritic cells, MP= Macrophages, NP=Neutrophils).

Supplementary Figure 4 Collected health data from the experimental lung metastasis studies. **(A)** The collected running distance of the exercising mice, shown as median of the groups (solid lines) with indication of the SD (transparent areas). Orange= EX group, light grey= Pre-EX group, light blue=Post-Ex group. **(B)** Average running distance of the exercising mice (analyzed with a Kruskal-Wallis test with Dunn’s multiple comparisons test). **(C)** Mean food intake prior to metastasis induction. **(D)** Mean food intake after metastasis induction. Parametric data was analyzed with a Welch's t test (Figure **C**, **D**). **(E)** Body weight of the mice at study start, metastasis induction, and at study end (Analyzed with a two-way ANOVA).
