# Supplementary figures and images for: Exercise boost after surgery improves survival in model of metastatic breast cancer
Source: Front Immunol. 2025 Feb 24;16:1533798. doi: 10.3389/fimmu.2025.1533798 (PMC11891249; doi:10.3389/fimmu.2025.1533798)

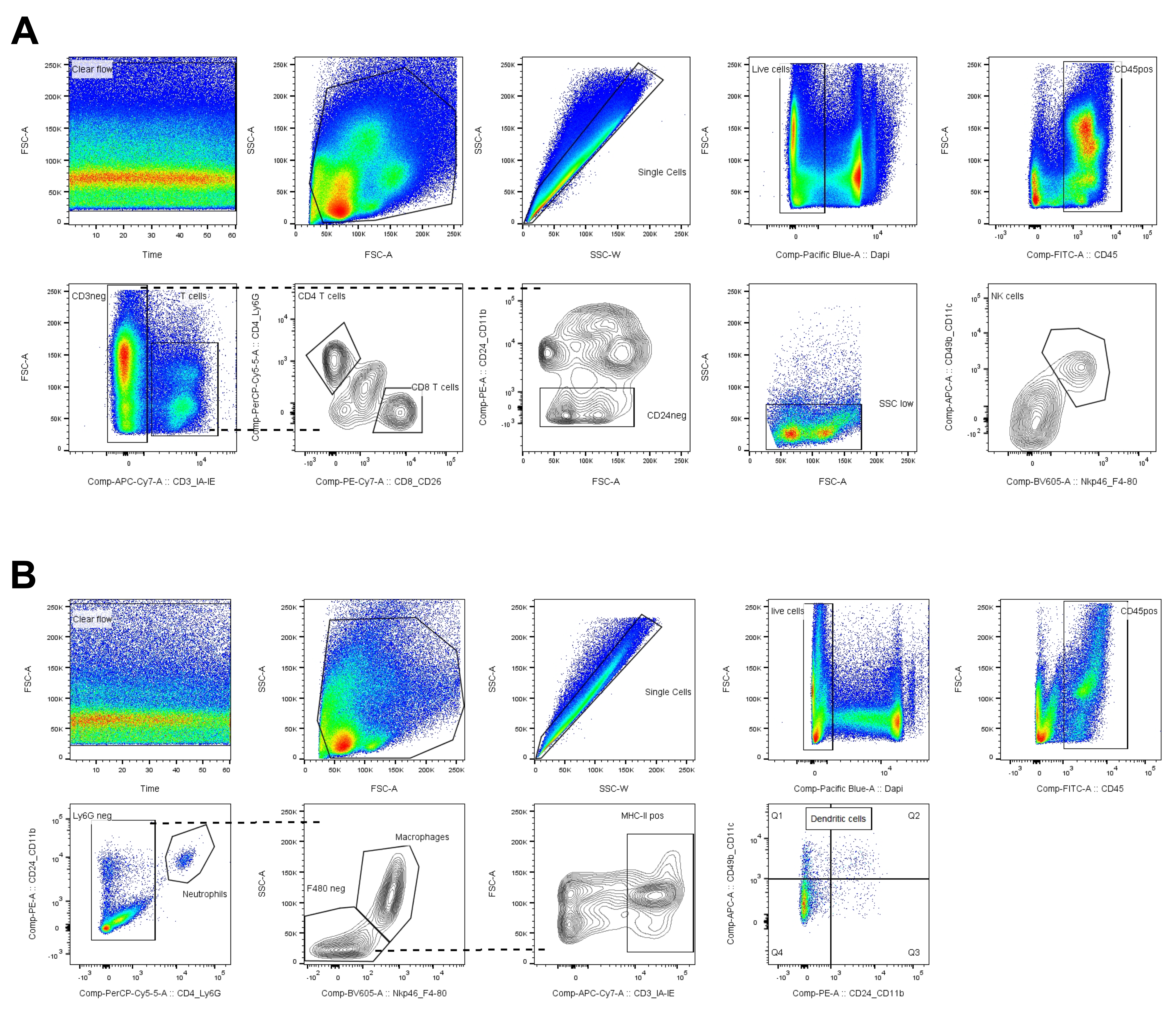

Supplement: Supplementary Figure 1 — Gating strategies for flow cytometry. (A) Gating strategy for panel 1. Cells were defined as T cells (CD3+), CD4 T cells (CD3+ CD4+), CD8 T cells (CD3+ CD8+), NK cells (CD3- CD24- SSClow CD49dim/hi Nkp46dim/hi). (B) Gating strategy for panel 2. Cells were defined as Neutrophils (Ly6G+), Macrophages (Ly6G- SSChi F4/80+) and Dendritic cells (Ly6G- F4/80- MHC-II+ CD11c+ CD11b+/-). [file Image1.tiff]

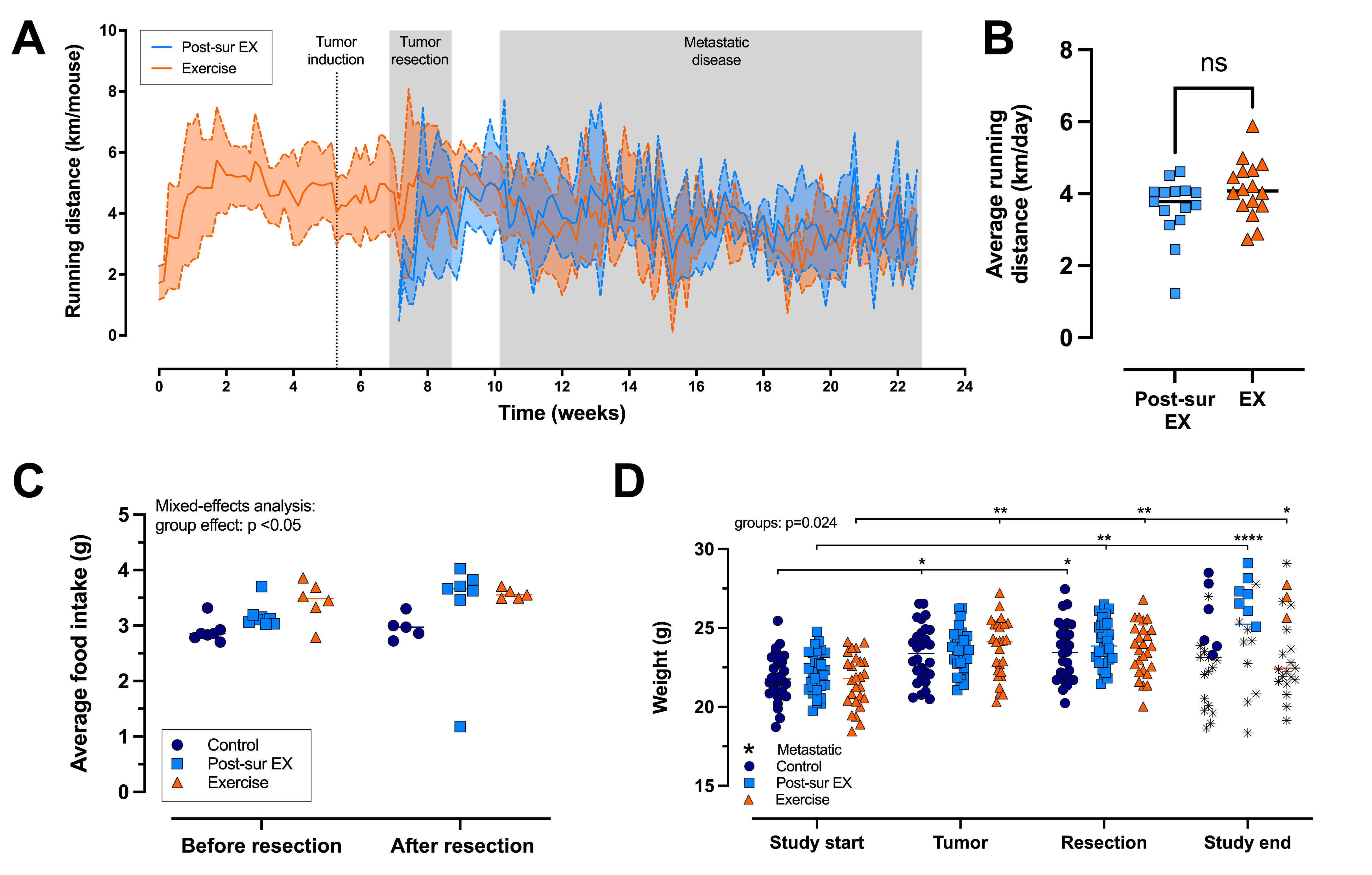

Supplement: Supplementary Figure 2 — Health data from the orthotopic tumor growth studies reveals no significant differences between the groups. (A) The collected running distance of the exercising mice, shown as median of the groups (solid lines) with indication of the SD (transparent areas) (orange = EX, light blue = Post-sur EX). (B) Average running distance of the exercising mice (C) Average food intake. (D) Body weight of the mice a study start, tumor inoculation (Tumor), tumor resection (Resection), and at study end/humane endpoints (Study end). [file Image2.tiff]

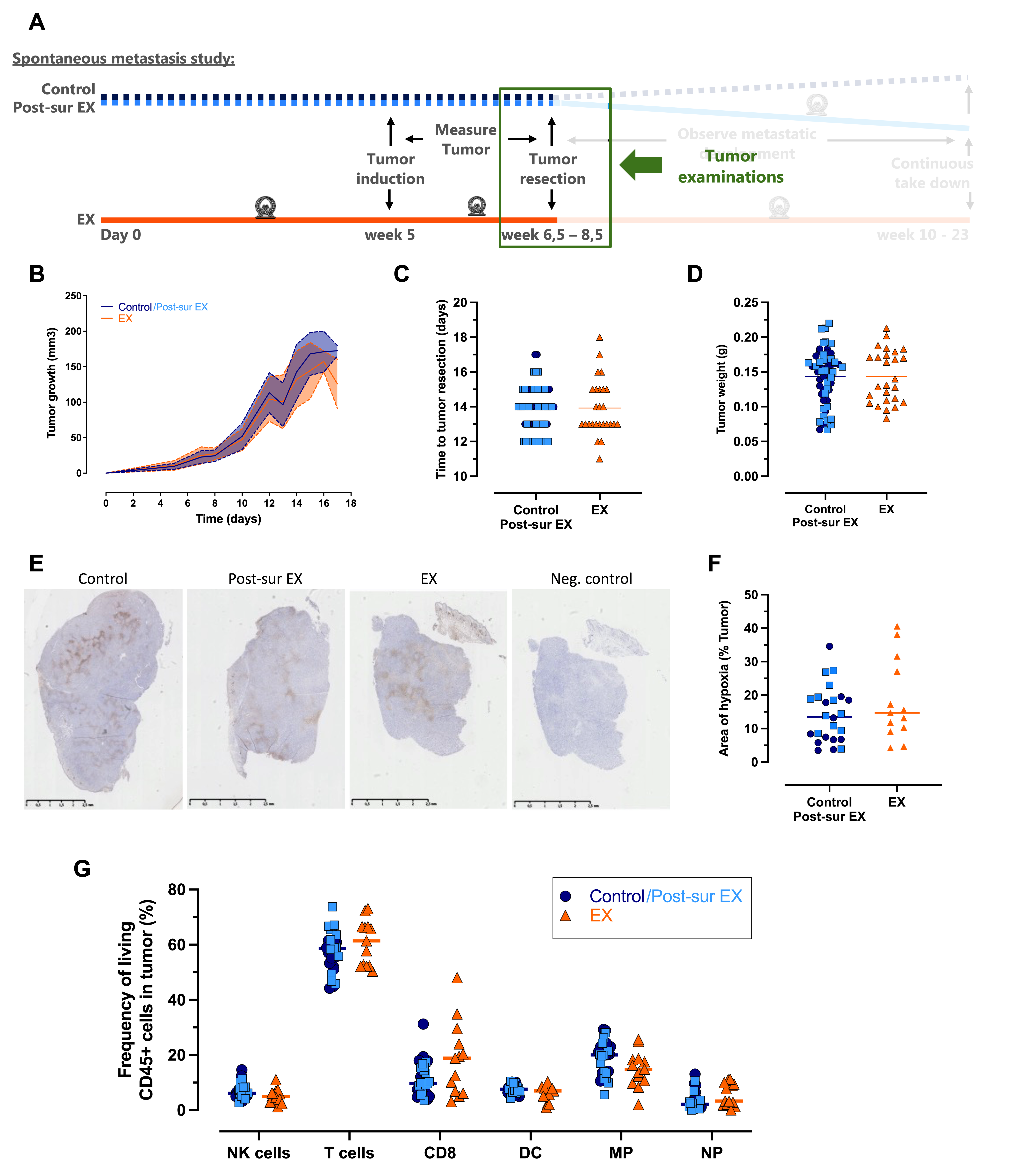

Supplement: Supplementary Figure 3 — Analysis of primary tumors reveals no significant differences between the groups in orthotopic model. (A) Visual representation of the experimental design and highlighting the focus of the data – i.e. examinations of resected tumors. (B) The tumor growth of mice with or without access to running wheels (Control/Post-sur EX= 58, EX=26). The solid line represents mean tumor growth, while the shaded area between the stippled lines constitutes the SD. Tumor growth data was analyzed with a nonlinear regression model using the exponential growth with log(population) equation, but not significant difference was detected. (C) Time from tumor inoculation to resection. (D) Weight of resected tumors. (E) Representative images of the degree of tumor hypoxia determined. (F) Percentage of tumor tissue affected by hypoxia (Control/Post-sur EX= 24, EX=13). (G) Flow cytometry analysis of the immune landscape in the resected tumors (Control/Post-sur EX= 28, EX=13). Parametric data was analyzed with an unpaired t-test (Figure C, G: T cells, MP) or a Welch's t test (Figure G: DC) and nonparametric data with a Mann Whitney test (Figure C, F, G: NK cells, CD8, NP). Tumor growth data was analyzed with a nonlinear regression model using the exponential growth with log(population) equation. (NK cells = natural killer cells, CD8 = CD8 positive T cells, DC= dendritic cells, MP= Macrophages, NP=Neutrophils). [file Image3.tiff]

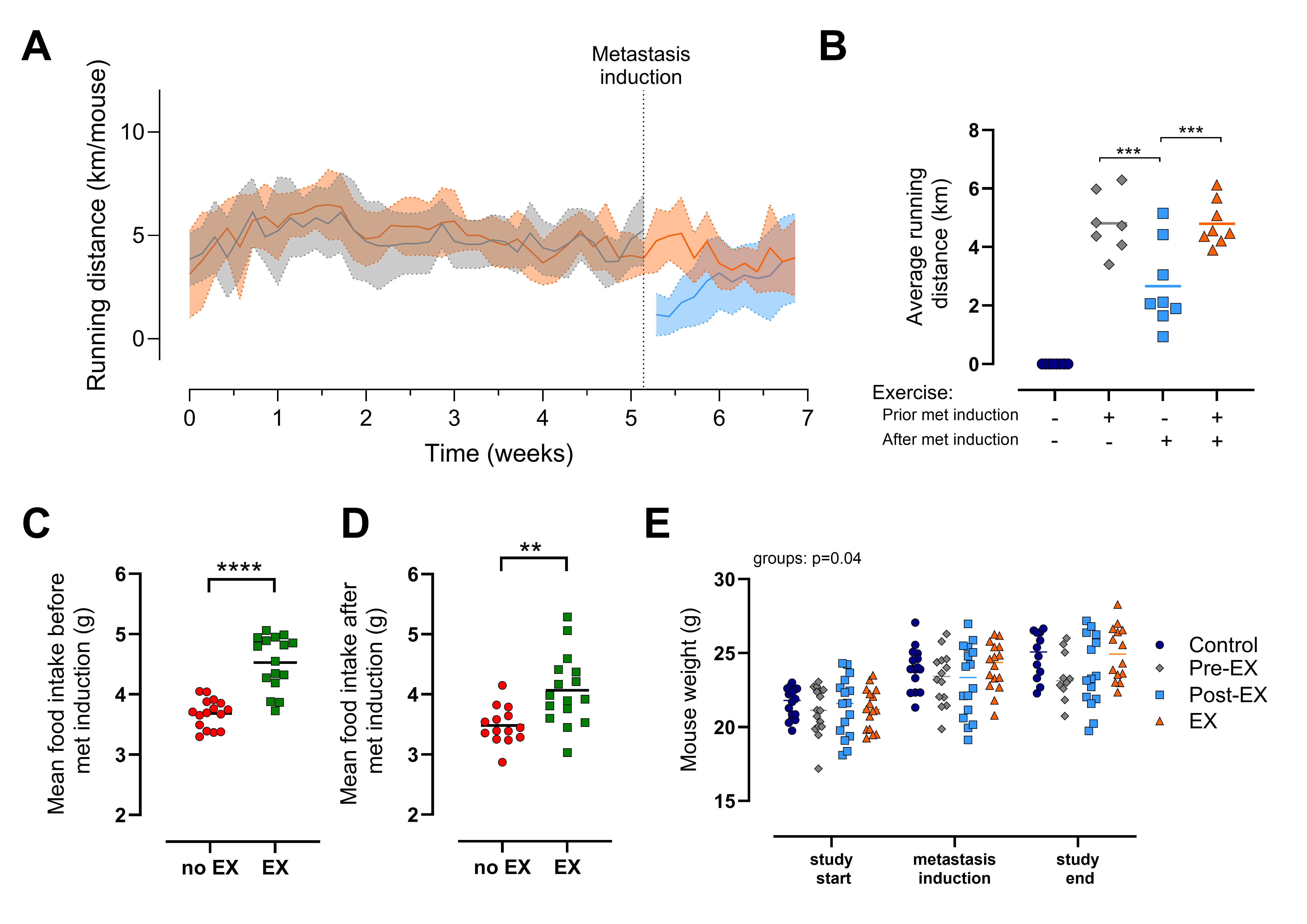

Supplement: Supplementary Figure 4 — Collected health data from the experimental lung metastasis studies. (A) The collected running distance of the exercising mice, shown as median of the groups (solid lines) with indication of the SD (transparent areas). Orange= EX group, light grey= Pre-EX group, light blue=Post-Ex group. (B) Average running distance of the exercising mice (analyzed with a Kruskal-Wallis test with Dunn’s multiple comparisons test). (C) Mean food intake prior to metastasis induction. (D) Mean food intake after metastasis induction. Parametric data was analyzed with a Welch's t test (Figure C, D). (E) Body weight of the mice at study start, metastasis induction, and at study end (Analyzed with a two-way ANOVA). [file Image4.tif]

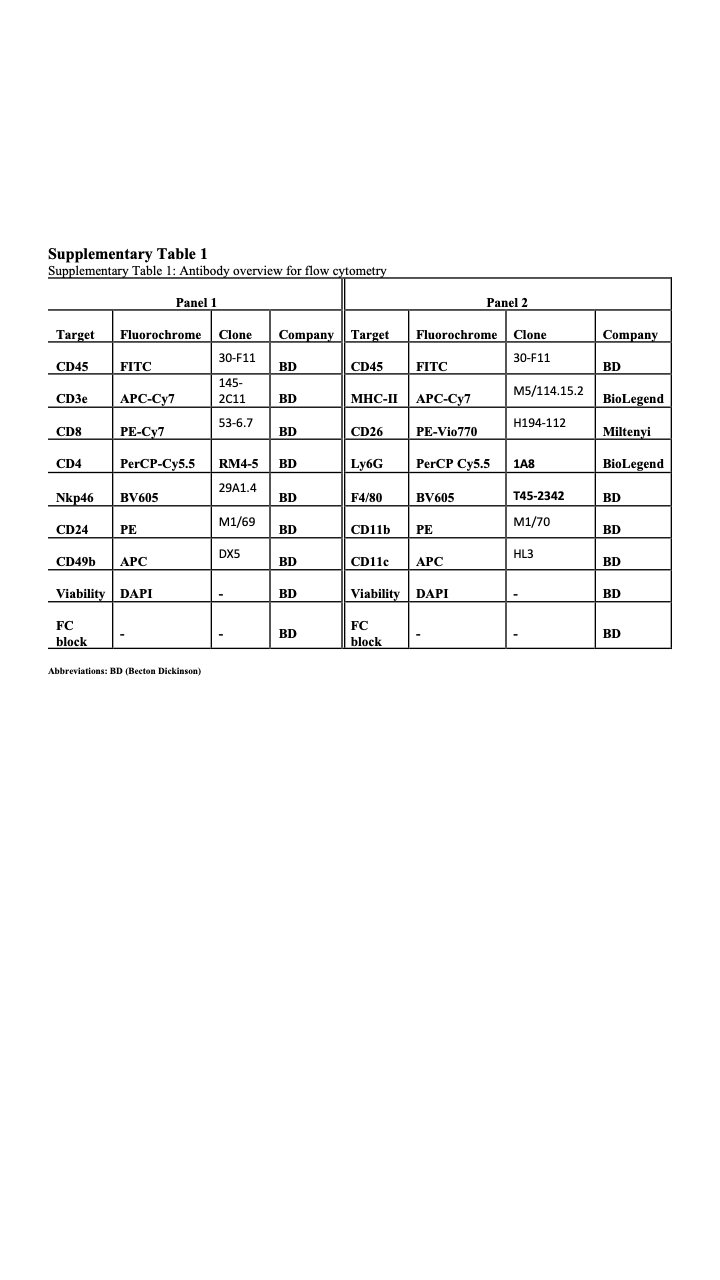

Supplement: Supplementary file 5 [file Image5.tiff]
